# Parasitoids of *Monochamus galloprovincialis* (Coleoptera, Cerambycidae), vector of the pine wood nematode, with identification key for the Palaearctic region

**DOI:** 10.3897/zookeys.251.3986

**Published:** 2012-12-18

**Authors:** Ricardo Petersen-Silva, Juli Pujade-Villar, Pedro Naves, Edmundo Sousa, Sergey Belokobylskij

**Affiliations:** 1 Instituto Nacional de Investigação Agrária e Veterinária, Quinta do Marquês, 2780–159 Oeiras, Portugal; 2 Departament de Biologia Animal, Facultat de Biologia, Universitat de Barcelona, Avda. Diagonal 645, 08026, Barcelona, Spain; 3 Museum and Institute of Zoology, Polish Academy of Sciences, Wilcza 64, Warszawa 00–679, Poland

**Keywords:** Braconinae, Ichneumonidae, parasitoids, *Monochamus galloprovincialis*, Cerambycidae, key to species

## Abstract

The parasitoid complex associated with *Monochamus galloprovincialis* (Olivier), vector of the pine wood nematode, is discussed. Four species of the family Braconidae and one Ichneumonidae were found associated with *Monochamus galloprovincialis* in Portugal, namely *Atanycolus denigrator* (Linnaeus), *Atanycolus ivanowi* (Kokujev), *Cyanopterus flavator* (Fabricius), *Doryctes striatellus* (Nees) (Braconidae), and *Xorides depressus* (Holmgren) (Ichneumonidae). *Atanycolus ivanowi*, *Atanycolus denigrator*, *Doryctes striatellus* and *Xorides depressus* are new species for Portugal fauna, and *Monochamus galloprovincialis* is recorded as a new host of *Xorides depressus*. A key for determination of the ichneumonoid parasitoids of the pine sawyer is provided for the Palaearctic fauna.

## Introduction

Worldwide, beetles of the genus *Monochamus* Dejean (1821) (Coleoptera; Cerambycidae) are the most important vectors of the pine wood nematode *Bursaphelenchus xylophilus* (Steiner and Buhrer 1934) (Nematoda, Tylenchida, Aphelenchoididae) (Linit 1988, 1990, Kishi 1995). This nematode is native to North America, where it’s not considered a primary pathogen of indigenous pines, although in countries where it has been introduced it is an important agent of mortality for susceptible pines. In Portugal, where the nematode has been present for over a decade now, the pine sawyer *Monochamus galloprovincialis* (Olivier 1795) is it’s sole vector (Sousa et al. 2001).

The pine sawyer *Monochamus galloprovincialis* is widely distributed in Europe (except in the United Kingdom, Ireland and Cyprus), and is also present in Caucasus, Russia, North Africa, China and Mongolia (Hellrigl 1971, Francardi and Pennacchio 1996). The *Monochamus* beetles do not breed on healthy trees, and are attracted only to stressed, dying or recently killed trees and freshly felled timber for egg laying (Linsley 1959, Hellrigl 1971, Linit 1987, Hanks 1999). Before the introduction of *Bursaphelenchus xylophilus* in Portugal, *Monochamus galloprovincialis* was considered a secondary forest pest and nothing was known about the most important aspects of its biology and ecology. In the rest of Europe there is also an absence of detailed studies on its biology, with the exception of the classic paper of Hellrigl (1971).

The most efficient way to control wilt disease is to decrease the population levels of the vector *Monochamus* beetles. However, the different methods to control these insects usually have success only in localized, small-dimension areas, but are difficult to implement at low cost and have reduced efficiency over large forested areas. In Portugal, the most important management and control strategy consists in the elimination of symptomatic trees during late autumn, winter and early spring, while the insect vector is inside the host as late-instar larvae or pupae. The vector’s populations can also be diminished during the beetle´s flight season with the use of traps baited with attractive lures (Naves et al. 2008).

Specific and efficient natural enemies (bio-control) would be an interesting and environmental-friendly option, but until now there are no adequate species for such control program (Naves et al. 2005). A few studies have already dealt with the parasitoids of this pine pest (Francardi and Pennacchio 1996, Francardi et al. 1998, Naves et al. 2005), although the information is scarce and disperse. In this paper we report on the diversity of parasitoids associated with *Monochamus galloprovincialis* in Portugal, their frequency and revise all previous information on ichneumonoids parasitoids of *Monochamus galloprovincialis* in the Palaearctic Region, resulting in a key for their identification.

## Material and methods

For the parasitoid surveys, two different approaches were employed:

I – Field surveys were made in five main pine regions in Portugal, selected by their different environmental characteristics, between April and October 2011. The areas where the study was made were:

Monção: (Lat: 42.075801, Lon: -8.517426)

Marinha Grande, Leiria: (Lat: 39.751677, Lon: -8.9977)

Comporta: (Lat: 38.35808, Lon: -8.772995)

Vale Feitoso, Idanha-a-Nova: (Lat: 40.064935, Lon: -6.987579)

In each location two dead *Pinus pinaster* Aiton, trees were felled, the wood sections colonized by *Monochamus galloprovincialis* were divided into 60 cm logs, and taken to the INIAV (Instituto Nacional de Investigação Agrária e Veterinária) laboratories in Oeiras to be stored in wood boxes prepared with a wire mesh. The boxes were completely covered with a black plastic, leaving two holes with transparent containers to collect emerged insects. Boxes were analyzed every two days for insects, which were collected and stored in alcohol for posterior identification. Additionally, the boxes were opened frequently for the evaluation of the content and to collect other emerged insects. The logs were kept inside the boxes until no emergences were registered for a period of two months, and then debarked and opened (with a vertical chain saw) to detect the hosts and life stages attacked by the parasitoids.

II – Complementary to the previous approach, artificial trap-trees were also prepared, consisting of living maritime pine trees which were cut into logs and given to adult *Monochamus galloprovincialis* to lay eggs under laboratory controlled conditions. Each log had a medium length of 40 cm and a medium diameter of 10 cm. subsequently, the logs were taken to the terrain to be colonized by parasitoids, and in each log a hole was made to allow passing a rope to hang them in the trees, at the branches height.

A total of 96 logs were divided according to the insect´s life stages (eggs, phloem larvae, xylem larvae and pupae), with 24 replicates each. Trials were made in Monção, Marinha Grande (Leiria), Comporta and Vimeiro, Alcobaça (Lat: 39.477811, Lon: –9.022316). In each location, six trap-trees were taken to the terrain four times during the year (pupae: April; eggs: July; phloem larvae: August; xylem larvae: October), in synchrony with the natural life cycle of the insect, and hanged in a healthy adult pine tree for a period of ten days. Subsequently, the logs were taken to the INIAV laboratory, and kept in wooden boxes (similarly to the previous experiment) to allow for emergencies. All emerged insects were identified and prepared to be photographed in the stereomicroscopy and environmental scanning electron microscopy (Serveis Científico-Tècnics de la Universitat de Barcelona). The field-emission gun environmental scanning electron microscope (FEI Quanta 200 ESEM) was used for high-resolution imaging without gold-coating with the purpose of not damaging the specimens.

All the collected material was stored in INRB Forestry Entomology Collection, Oeiras, Portugal. The insects collected with the trap-tree were labeled “Artificial” and the ones from the dead trees were labeled “Natural”. Terminology employed in the key for morphological features, sculpture and measurements as well as wing venation nomenclature follows Belokobylskij and Maeto (2009).

## Results

Besides *Monochamus galloprovincialis*, the following insects emerged from the wood and logs: *Arhopalus* sp. (Coleoptera: Cerambycidae), *Orthotomicus erosus* (Wollaston 1857) (Coleoptera: Scolytidae), *Thanasimus formicarius* (Linnaeus 1758) (Coleoptera: Cleridae), *Sirex noctilio* Fabricius (1793) (Hymenoptera: Siricidae), and some species of the family Anobiidae. Other bark beetles (Scolytidae) were also present in the dead tree material, although they were not analyzed.

No parasitoids emerged from the trap trees with eggs, xylem larvae and pupae of *Monochamus galloprovincialis*. Parasitism was only found in the sub-cortical larvae, corresponding to the host´s first instars. A total of 27 specimens, belonging to five species, were recovered solely from Marinha Grande and Vale Feitoso, seven of which (all *Cyanopterus flavator*) from the trap-trees, while the remaining species were all obtained from the dead trees. *Cyanopterus flavator* (Fabricius)and *Atanycolus ivanowi* (Kokujev)were found in Vale Feitoso, and in Marinha Grande the following ichneumonids and braconids were recovered: *Atanycolus denigrator* (L.); *Cyanopterus flavator*; *Doryctes striatellus* (Nees) and *Xorides depressus* (Holmgren).

By far, *Cyanopterus flavator* was the most abundant species with a total of 15 specimens from Marinha Grande and Vale Feitoso. Cocoons of this species were found in the xylem galleries of *Monochamus galloprovincialis*, alongside with mandibles of the dead larvae.

The other cocoons found were in the inner bark associated with the larval galleries of the pine sawyer. The number of cocoons found matches exactly the number of parasitoids obtained from this surveys, and no other cocoons were found parasitizing any of the species previously mentioned. The parasitized species emerged between May and September under laboratory conditions and the precise dates are recorded in the labels of each specimen.

The following hymenoptera emerged from the wood, with both Braconidae (4) and Ichneumonidae (1):

### Family Braconidae

#### Atanycolus denigrator

(Linnaeus 1758)

http://species-id.net/wiki/Atanycolus_denigrator

Figures 7a, e

##### Material examined.

Portugal: 1 female, “Leiria, 1/6/2011”, “Ensaio Pupas Natural”, “Col. Estação Florestal Nacional”; 1 male, “Leiria, 1/6/2011”, “Ensaio Pupas Natural”, “Col. Entomologica, est. Florestal”.

##### Distribution.

**Palaearctic**: Austria, Bulgaria, China, Croatia, former Czechoslovakia, Finland, France, Germany, Greece, Hungary, Israel, Italy, Kazakhstan, Korea, Mongolia, Norway, Poland, Russia, Sweden, Switzerland, Turkey, United Kingdom. **Afrotropical**: Niger (Yu et al. 2005, Wang et al. 2009). This species is here recorded for Portugal for the first time.

##### Hosts.

*Anthaxia morio* Fabricius, *Chrysobothris chrysostigma* Linnaeus, *Chrysobothris solieri* Laporte & Gory, *Lampra rutilans* (Fabricius), *Poecilonota variolosa* (Paykull) (Buprestidae); *Acanthocinus aedilis* Linnaeus, *Acanthocinus griseus* (Fabricius), *Arhopalus syriacus* (Reitter), *Monochamus galloprovincialis* (Oliver), *Monochamus sutor* (Linnaeus), *Rhagium indagator* Fabricius, *Rhagium inquisitor* Linnaeus, *Rhagium mordax* (Degeer), *Saperda populnea* (Linnaeus), *Tetropium castaneum* (Linnaeus), *Tetropium fuscum* (Linnaeus), *Tetropium gabrieli* Weise (Cerambycidae); *Ips sexdentatus* (Boerner) (Scolytidae) (Yu et al. 2005, Wang et al. 2009).

##### Biology.

*Atanycolus denigrator* is an ectoparasitoid of *Monochamus galloprovincialis* attacking *Pinus pinaster*. The species was found parasitizing the first larval instars under the bark of the tree.

##### Remarks.

*Atanycolus denigrator* was already recorded in Italy as parasitoid of *Monochamus galloprovincialis* (Campadelli and Scaramozzino 1994). We additionally studied this reared Italian material in Hungarian Natural History Museum in Budapest (1 female, “Italia, Ravenna, 14.IV.1992, Campadelli”, “Pinus picea”, “ex larva *Monochamus galloprovincialis* Ol., 21.IV.1992”, “*Atanycolus* ♀ *denigrator* L. det. Papp J. 2000”; 1 female, same first label, but “21.IV.1992”, second and third labels are the same ones) and confirmed present determination.

#### Atanycolus ivanowi

(Kokujev 1898)

http://species-id.net/wiki/Atanycolus_ivanowi

Figures 4c 5b 6d

##### Material examined.

Portugal: 4 females, “Vale Feitoso II, Maio 2011”, “Col. Entomologica, est. Florestal”; 1 female, same labels, but 12.VI.2011; 1 male, “Vale Feitoso II, Maio 2011”.

##### Distribution.

**Palaearctic**: Armenia, Austria, Azerbaijan, Croatia, Czechia, Finland, France, Germany, Greece, Hungary, Italy, Japan, Kazakhstan, Russia, Slovakia, Switzerland, Tajikistan, Turkmenistan, Ukraine, Uzbekistan (Yu et al. 2005) and Turkey (Bolu et al. 2009). This species is here recorded for Portugal for the first time.

##### Hosts.

*Anthaxia aurulenta* (Fabricius), *Anthaxia deaurata* (Gmelin), *Chrysobothris solieri* (Laporte & Gory), *Ovalisia mirifica* (Mulsant), *Melanophila picta decastigma* (Fabricius); *Sphenoptera tappesi* Marseul (Buprestidae); *Arhopalus syriacus* (Reitter), *Stictoleptura rubra* (Linnaeus), *Monochamus galloprovincialis* (Olivier), *Tetropium fuscum* (Fabricius), *Tetropium gabrieli* Weise (Cerambycidae) (Yu et al. 2005, Bolu et al. 2009).

##### Biology.

*Atanycolus ivanowi* was found to be an ectoparasitoid of first larval stages of *Monochamus galloprovincialis* living under the bark of *Pinus pinaster*.

##### Remark.

*Monochamus galloprovincialis* was already recorded by Campadelli and Scaramozzino (1994) as a host of *Atanycolus ivanowi* in Italy.

#### Cyanopterus flavator

(Fabricius 1793)

http://species-id.net/wiki/Cyanopterus_flavator

Figures 1d 7b, d, g

##### Material examined.

Portugal: 1 female, “Leiria, 14/6/2011”, “Ensaio Pupas Natural”, “Col. Entomologica, est. Florestal”, “13”; 1 female, same labels, 17.VI.2011, N 12. “Leiria, Larvas Artificial”: 1 female, N 26; 1 female, N 27. “Leiria, Posturas Artificial”: 1 female, 31.VIII.2011, N 25; 1 female, N 27. “Leiria, Ensaio, Posturas Artificial”: 1 male, 31.VIII.2011, N 21. “Vale Feitoso”: 1 female, N 9; 1 female, N 10; 1 male, N 11; 1 female, N 14; 1 female, N 15; 1 female, N 16; 1 female, N 17.

##### Distribution.

**Palaearctic**: Algeria, Croatia, Cyprus, former Czechoslovakia, Finland, France, Germany, Greece, Hungary, Israel, Italy, Japan, Kazakhstan, Korea, Latvia, Morocco, Netherland, Poland, Romania, Russia, Spain, Switzerland, Syria, Tunisia, Ukraine, United Kingdom, former Yugoslavia (Yu et al. 2005) and Portugal (Naves et al. 2005).

##### Hosts.

*Bostrichus capucinus* (Linnaeus) (Bostrichidae); *Acanthocinus griseus* (Fabricius), *Acanthoderes clavipes* (Schrank), *Hesperophanes pallidus* (Olivier), *Monochamus galloprovincialis* (Olivier), *Monochamus sartor* (Fabricius), *Morimus asper* (Sulzer), *Phymatodes testaceus* (Linnaeus), *Pogonochaerus fasciculatus* (Degeer), *Pogonochaerus hispidus* (Linnaeus), *Rhagium inquisitor* (Linnaeus), *Saperda scalaris* (Linnaeus) (Cerambycidae) (Yu et al. 2005), and *Monochamus rosenmulleri* (Cederhjelm) (Watanabe 1937).

##### Biology.

The biology of this parasitoid is poorly known, but in this study all the specimens emerged from cocoons from the xylemic galleries of *Monochamus galloprovincialis*, which were not completely sealed with frass, as it is normal. Considering the length of the ovipositor of *Cyanopterus flavator*, it is apparent that only first larval instars of *Monochamus galloprovincialis* (found beneath the bark) are parasitized, which subsequently enter the wood carrying the parasitoid. Only the mandibles of the host larvae were found in galleries with cocoons.

##### Remark.

*Monochamus galloprovincialis* as a host of *Cyanopterus flavator* was already recorded by Campadelli and Scaramozzino (1994) for Italy and Naves et al. (2005) for Portugal.

#### Doryctes striatellus

(Nees 1834)

http://species-id.net/wiki/Doryctes_striatellus

Figures 1b 4b, d 5a

##### Material examined.

Portugal: 1 female, “Leiria, 12/8/11”, “Ensaio Pupas Natural”, 1 male, same labels, but 9.VIII.2011; 1 male, same label, but 29.VII.2011.

##### Distribution.

**Palaearctic**: Austria, Belgium, Bulgaria, China, Czechia, Finland, France, Germany, Hungary, Italy, Japan, Lithuania, Poland, Russia, Slovakia, Sweden, Switzerland, Ukraine, United Kingdom (Yu et al. 2005). This species is here recorded for Portugal for the first time.

##### Hosts.

*Ernobius mollis* (Linnaeus), *Dorcatoma dresdensis* Herbst (Anobiidae); *Anthaxia quadripunctata* (Linnaeus), *Phaenops cyanea* (Fabricius), *Phaenops guttulata* (Gebler) (Buprestidae); *Acanthocinus aedilis* (Linnaeus), *Agapanthia* sp., *Callidium* sp., *Callidium violaceum* (Linnaeus), *Clytus* sp., *Exocentrus lusitanus* (Linnaeus), *Mesosa curculionoides* (Linnaeus), *Molorchus minor* (Linnaeus), *Monochamus galloprovincialis* (Olivier), *Monochamus sutor* (Linnaeus), *Phymatodes pusillus* (Fabricius), *Phymatodes testaceus* (Linnaeus), *Poecilium alni* (Linnaeus), *Pogonocheru*s sp., *Pogonocheru hispidus* (Linnaeus), *Rhagium inquisitor* (Linnaeus), *Semanotus undatus* (Linnaeus), *Stenostola ferrea* (Schrank), *Tetropium castaneum* (Linnaeus), *Tetropium gabrieli* Weise, *Tetropium fuscum* (Fabricius), *Tetropium gracilicorne* Reitter (Cerambycidae); *Pissodes harcyniae* (Herbst), *Pissodes notatus* (Fabricius), *Rhynchaenus fagi* (Linnaeus), *Rhynchaenus pilosus* (Fabricius), *Rhynchaenus quercus* (Linnaeus), *Rhynchaenus testaceus* (Müller), *Magdalis violacea* (Linnaeus), *Magdalis rufa* (Germar), *Tachyerges salicis* (Linnaeus), (Curculionidae); *Hylurgops palliatus* (Gyllenhal), *Ips typographus* (Linnaeus), *Ips sexdentatus* (Boerner), *Ips subelongatus* Motschulsky, *Pityogenes bidentatus* (Herbst), *Tomicus piniperda* (Linnaeus) (Scolytidae); *Xyphidria prolongata* (Geoffroy) (Xyphidriidae) (Yu et al. 2005).

##### Remark.

This species was already recorded in Italy on the name *Doryctes mutillator* (Thunberg) as parasitoid of *Monochamus galloprovincialis* (Campadelli and Scaramozzino 1994).

### Family Ichneumonidae

#### Xorides depressus

(Holmgren 1860)

http://species-id.net/wiki/Xorides_depressus

Figures 1c 3b, e

##### Material examined.

Portugal: 1 female, “Leiria, Pupas Natural”, 19.VII.2011; 1 female, “Leiria, 29/7/11, Ensaio Pupas Natural”; 1 female, N 19.

##### Distribution.

**Palaearctic**: Austria, former Czechoslovakia, Finland, France, Germany, Hungary, Latvia, Poland, Romania, Russia, Spain, Sweden (Yu et al. 2005). This species is here recorded for Portugal for the first time.

##### Hosts.

*Melanophila cyanea* (Fabricius) (Buprestidae); *Nothorhina punctata* (Fabricius) (Cerambycidae) (Yu et al. 2005). *Monochamus galloprovincialis* (Olivier) is a new host of *Xorides depressus* from Portugal.

## Discussion

Considering the literature data and information presented in this study, the following key identifies the species of parasitoids attacking this pine sawyer in the Palaearctic Region. Only species that are reliably confirmed as parasitoids of *Monochamus galloprovincialis* were considered for the key. Some species of parasitoids were excluded from this list and discussion about this decision is present in the final section. A key to species of Ichneumonidae and Braconidae parasitoids of *Monochamus galloprovincialis* is presented:

**Table:** 

1	Second recurrent vein of fore wing present (Figure 1a). In hind wing, second longitudinal cubital vein always present and arising near middle of nervellus. Second and third metasomal tergites movable, not fused (Figures 1c, 2g) (Fam. Ichneumonidae). – Spiracles of first metasomal tergite placed on or before its middle (Figure 2e–f)	2
–	Second recurrent vein of fore wing absent (Figuress 1b, 4e, 5d). In hind wing, second longitudinal cubital vein absent. Second and third metasomal tergites immovable, fused (Figures 1d, 5a–c) (Fam. Braconidae)	4
2	First metasomal sternite distinctly separated from tergite, this tergite with glymma (Figure 2e), and/or propodeum without transverse basal carina (Figure 2a). Claws of leg in female with teeth or basal lobe (Figure 2c) (Pimplinae) – Second metasomal tergite with pair of oblique furrows running from almost middle of its base to spiracles (Figure 2g). Lower valva of ovipositor apically with lateral lobes covered partly upper valva; dorsal lobe of lower valva with six–seven furrows. Ovipositor sheath 1.2–1.3 times longer than body. Body entirely black (including corner of pronotum); tegula brownish yellow; pterostigma dark; legs red, hind tibia and tarsus brownish red. Body 10.0–22.0 mm	*Dolichomitus tuberculatus* (Geoffroy)
–	First metasomal sternite fused with tergite, this tergite without glymma (Figure 2f); propodeum always at least with track of transverse basal carina (Figure 2b). Claws of leg in female simple, without teeth or basal lobe (Figure 2d) (Xoridinae)	3
3	Hind femur wide, with strong median ventral tooth (Figure 3a). Temple distinctly punctuate (Figure 3d). – Middle tibia posteriorly without deep oblique groove. Second metasomal tergite transverse and finely punctuate. Ovipositor sheath about as long as body. Body blackish; flagellum of antenna rufous; legs mainly reddish, coxae blackish. Body length 5.0–9.0 mm	*Odontocolon quercinum* (Thomson)
–	Hind femur narrow, without ventral tooth (Figure 3b). Temple finely obliquely striate (Figure 3e). – First metasomal tergite beyond middle without dorsal longitudinal carinae, about twice longer than wide (Figure 1c). Ovipositor sheath about as long as body. Antennae brownish, without white band; hind leg brownish, but tarsus rufous; first and second metasomal tergites reddish. Body length 6.0–11.0 mm	*Xorides depressus* (Holmgren)
4	Hypoclypeal depression absent; middle of ventral margin of clypeus situated close to upper level of mandibles (Figure 4a). Brachial cell of fore wing open in distal posterior part, brachial vein absent. Second radiomedial cell of fore wing short (Figure 4e). (Euphorinae). – Second metasomal tergite striate (Figure 3c). Body mainly black. Body length 6.0–8.0 mm	*Meteorus corax* Marshall
–	Hypoclypeal depression deep and wide; middle of ventral margin of clypeus situated distinctly above upper level of mandibles (Figure 4b). Brachial cell of fore wing closed by brachial vein in distal posterior part. Second radiomedial cell of fore wing usually long (Figure 1b)	5
5	Occipital and prepectal carinae present (Figure 4d). First tergite with distinct dorsope and without median area delineated by furrows (Figure 5a). Recurrent vein of hind wing present. Submedial cell of hind wing long. (Doryctinae). Body length 3.0–6.5 mm	*Doryctes striatellus* (Nees) (*Doryctes mutillator* auct.)
–	Occipital and prepectal carinae absent (Figure 4c). First tergite without dorsope and with median area delineated by furrows (Figures 1d, 5b, c, 6c, 7a-c). Recurrent vein of hind wing absent. Submedial cell of hind wing short (Figure 5d). (Braconinae)	6
6	Pedicel of antenna almost as long as first flagellar segment. First and second flagellar segments not longer than median segments of flagellum and concave below (Figure 6a). Second metasomal tergite without mediobasal triangle area (Figure 5c). – Ovipositor short, its sheath 1.0–1.3 times as long as metasoma, 0.60–0.65 times as long as fore wing. Second metasomal tergite about as long as third tergite, without or with fine oblique lateral furrows (Figure 5c). Body length 2.5–5.0 mm	*Coeloides sordidator* Ratzeburg
–	Pedicel of antenna distinctly shorter than first flagellar segment. First and second flagellar segments longer than median segments of flagellum and not concave below (Figures 6b, 7d). Second metasomal tergite usually with mediobasal triangle area separated by furrows (Figures 5b, 7a, c)	7
7	Second metasomal tergite without mediobasal triangle area separated by furrows (Figure 6c). Upper valva of ovipositor enlarged, distinctly larger than lower valva. Antenna setiform, longer than body. Body crimson-red with black spots. Body length 5.0–12.0 mm	*Iphiaulax impostor* (Scopoli)
–	Second metasomal tergite usually (except *Cyanopterus flavator*) with mediobasal triangle area separated by furrows (Figures 5b, 7a, c). Upper valva of ovipositor not enlarged, not larger than lower valva. Antenna more or less filiform, not longer than body. Body never crimson-red, usually black with yellowish brown spots or areas on head and always on most part of metasoma	8
8	Scape of antenna with strong basal constriction and with apical collar. Pedicel distinctly projected behind scape (Figures 6d, 7e, f). Furrow between antennal socket and eye present (Figure 7f)	9
–	Scape of antenna without basal constriction and without apical collar. Pedicel weakly projected behind scape (Figures 6b, 7d). Furrow between antennal socket and eye absent (Figure 7g)	11
9	Second–fourth metasomal tergites of female coarsely rugose-striate at least medially (Figure 5b). Head often more or less depressed dorso-ventrally (Figure 6d). Body length 5.0–9.0 mm	*Atanycolus ivanowi* (Kokujev)
–	Second–fourth metasomal tergites of female smooth (except sculptured furrows) (Figure 7a), rarely second tergite partly with rugosity. Head never depressed dorso-ventrally	10
10	Head mainly brownish yellow or light reddish brown, only dorsally black and usually in large wedge-shaped black spot. Body length 7.0–10.0 mm	*Atanycolus genalis* (Thomson) (*Atanycolus initiator* auct.)
–	Head mainly black, sometimes paler only near base of mandible, always with reddish stripes along inner side of eye. Body length 5.0–9.0 mm.	*Atanycolus denigrator* (Linnaeus)
11	Ventral margin of scape (lateral view) not shorter than dorsal margin (Figure 7d). Second tergite without basomedian area delineated by furrow (Figure 7b). Metasoma brownish yellow, behind first tergite entirely smooth. Wings strongly infuscate. Body length 6.0–10.0 mm	*Cyanopterus (Cyanopterus) flavator* (Fabricius)
–	Ventral margin of scape (lateral view) shorter than dorsal margin (Figure 6b). Second tergite with distinct basomedian area delineated by sculptured furrow (Figure 7c). Metasoma mainly dark brown or black, behind first tergite sculptured in furrows and suture. Wings faintly infuscate. Body length 4.0–5.0 mm	*Cyanopterus (Ipobracon) tricolor* (Ivanov)

With the exception of *Atanycolus ivanowi* collected from Vale Feitoso, all the other species were collected in Marinha Grande. This location is near Portugal´s oldest managed pine forest, in a pine stand with about 1700 square km and which was first planted in the XIII century. This stable and managed environment may have created favorable conditions for the establishment of a diverse entomofauna in the region. In fact, the larger number of parasitoids found in the region, and the low population levels of the vector insect suggest that *Monochamus galloprovincialis* may be locally well controlled by its natural enemies. Further studies in the Leiria pine stand should confirm this hypothesis.

There is no obvious reason for the absence of parasitoids in the other sampled locations, although factors such as the local density of *Monochamus galloprovincialis* (and other insect hosts), and differences in the local edapho-climatic conditions may explain the absence of the natural enemies.

Despite *Atanycolus* genus being the most diverse,* Cyanopterus* is the genus were the most specimens were found. Each parasitoid was reared from one specific location, except *Cyanopterus* specimens which were found in two very distanced sites, which present completely different edapho-climatic conditions.

According to Watanabe (1937), the cocoons of *Cyanopterus flavator* occurred in the trunk of *Picea jezoensis* Siebold et Zuccarini shut in by a thick corky lid at the end of the tunnel made by the larva of *Monochamus rosenmulleri*, a conclusion which completely supports the suggested hypothesis for the parasitizing activity in Portugal.

Worldwide and including this study, there is now a total of 14 species of parasitoids associated with *Monochamus galloprovincialis*, being six Ichneumonidae and eight Braconidae. Previous reliable records (confirmed rearing from the larvae of *Monochamus galloprovincialis*) in the literature include references from Portugal (Naves et al. 2005), Italy (Campadelli and Scaramozzino 1994), and Siberia (Tobias and Belokobylskij 2000), among other locations (Kenis and Hilszczanski 2004, Tobias et al. 1986; Yu et al. 2005). Although not detected in this study, other groups, such as the braconids of the subfamily Helconinae (namely species of *Helcon* Nees 1812 and *Helconidea* Viereck 1914), will also likely parasitized larvae of *Monochamus galloprovincialis* as they have been found to develop in larvae of other *Monochamus* species (Tobiaset al. 1986, Yu et al. 2005).

Other records are more dubious and need further confirmation. Among these, three records of Ichneumonidae are possibly erroneous, namely *Rhyssa persuasoria* (Linnaeus) (Pimplinae),* Perithous divinator* (Rossi) (Pimplinae) and *Stenarella domator* (Poda) (Cryptinae). The first species is a specialized parasitoid of Siricidae larvae (Yu et al. 2005), and its rearing from Cerambycidae is probably inaccurate. Likewise, the other two species are specialized parasitoids of vespoid and sphecoid wasps (Kasparyan 2010), and their associations with Cerambycidae is quite doubtful. Therefore, in the identification key only three ichneumonids were included, namely *Odontocolon quercinum* (Thomson), *Xorides depressus* (Holmgren) and *Dolichomitus tuberculatus* (Geoffroy). On the other hand, as all species of Braconidae were directly reared from *Monochamus galloprovincialis*, they were included in our key.

Despite the relatively high diversity of parasitoids associated with *Monochamus galloprovincialis* worldwide, all species are mainly idiobiont ectoparasitoids (except *Monochamus corax*) and seem to be generalists attacking a vast array of other insects living in dead and dying trees. *Cyanopterus flavator*, which had already been found parasitizing young larval stages (Naves et al. 2005), appears to be the most frequent and promising candidate for studies aiming the biological control of the pine sawyer, despite its generalist habits. As mentioned, the disperse distribution of *Cyanopterus* can be considered as a major adaptation two the diverse edapho-climatic conditions characteristic for Portugal. Other options, such as the introduction of exotic natural enemies would create new parasite-host interactions, which usually offer greater changes of success for biological control than the promotion of already established associations (Hokkanen and Pimentel 1984). Nevertheless, such measures require rigorous pre-release risk assessment of the economic and environmental costs and benefits of the introduction, to evaluate its potential effectiveness, host specificity, acclimatisation and viability for mass-production (van Lenteren et al. 2006).

Detailed studies on the effect of the parasitoid guild found in Portugal on the pine sawyer´s population and the suitability of the species for biological control are being planned, with the final objective of eventually establishing an integrated bio-control program against the vector of the pine wilt disease in Europe.

## Figure plates

**Figure 1.**
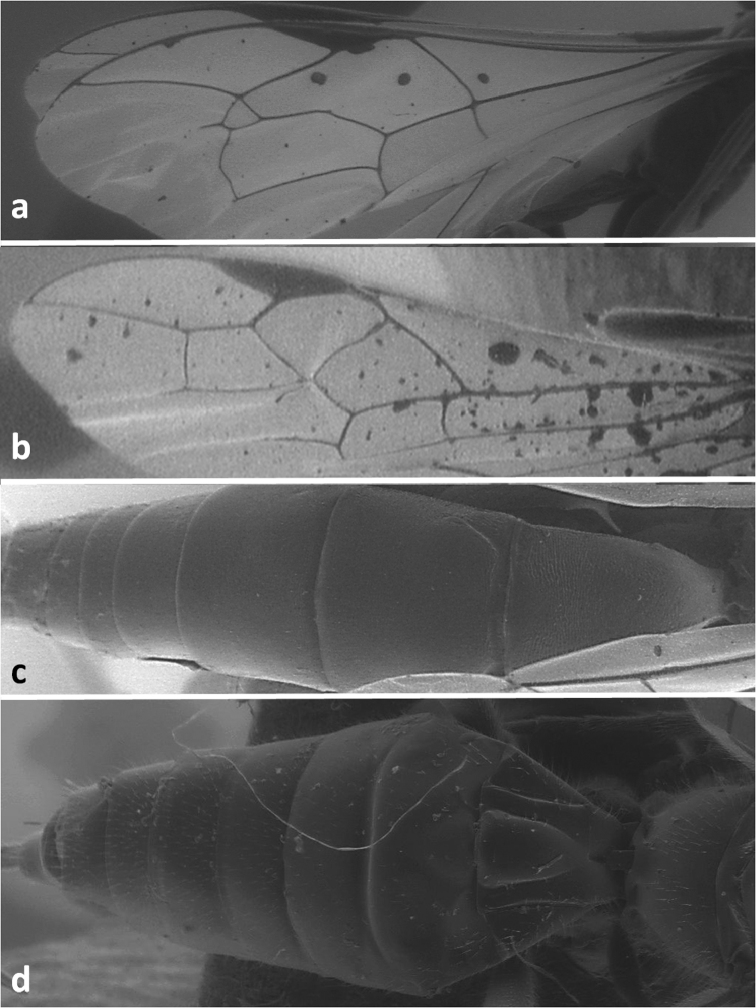
Forewing of *Dolichomitus tuberculatus*(**a**) and *Doryctes striatellus* (**b**); metasoma in dorsal view of *Xorides depressus* (**c**) and *Cyanopterus flavator* (**d**).

**Figure 2.**
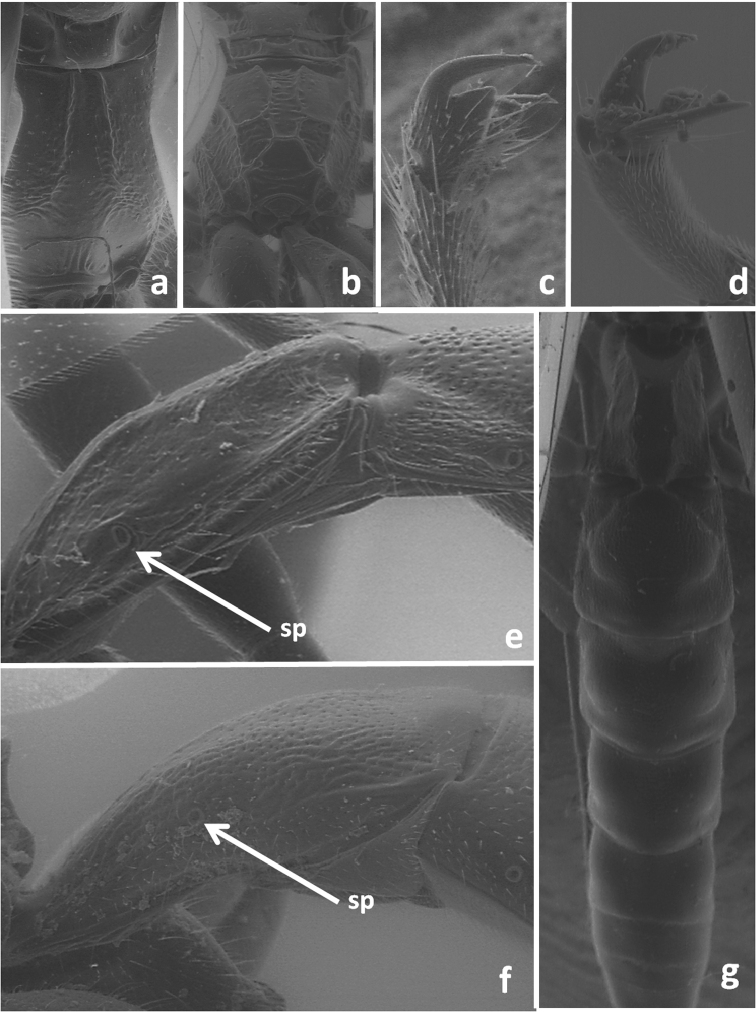
Propodeum in dorsal view of *Dolichomitus tuberculatus* (**a**) and *Odontocolon quercinum* (**b**); tarsal claws of *Dolichomitus tuberculatus* (**c**) and *Odontocolon quercinum* (**d**); first metasomal tergite in lateral view of *Dolichomitus tuberculatus* (**e**) and *Odontocolon quercinum* (f); dorsal view of metasoma of *Dolichomitus tuberculatus* (**e**); sp – spiracle.

**Figure 3.**
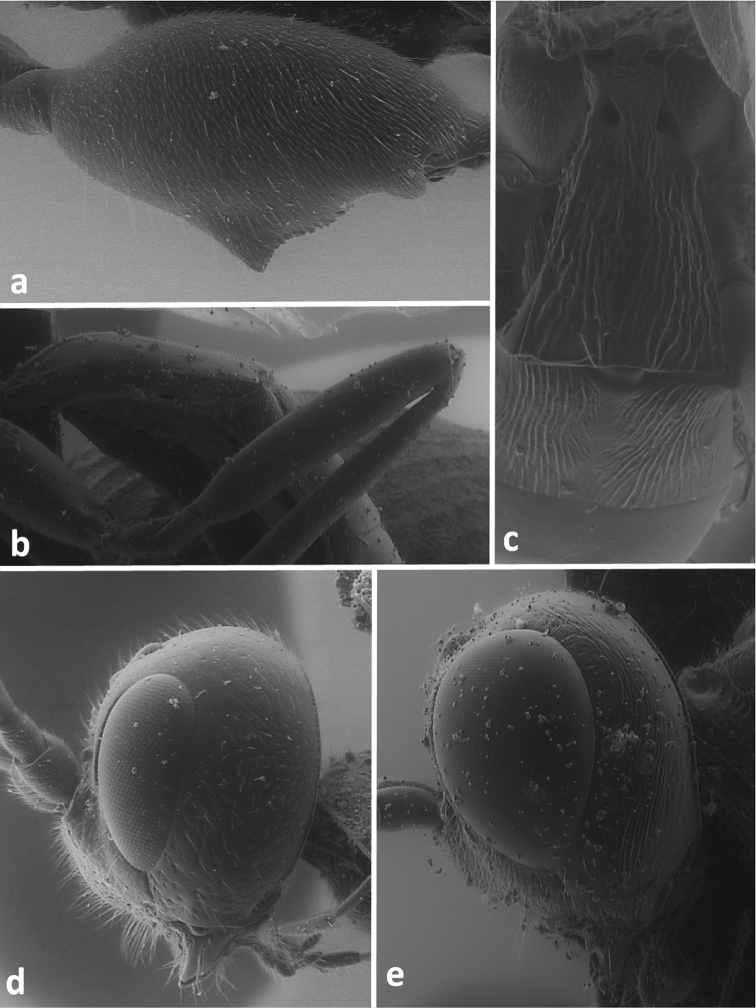
Lateral view of hind femur of *Odontocolon quercinum* (**a**) and *Xorides depressus* (**b**); first and second metasomal tergites in dorsal view of *Meteorus corax* (**c**);head in lateral view of *Odontocolon quercinum* (**d**) and *Xorides depressus* (**e**).

**Figure 4.**
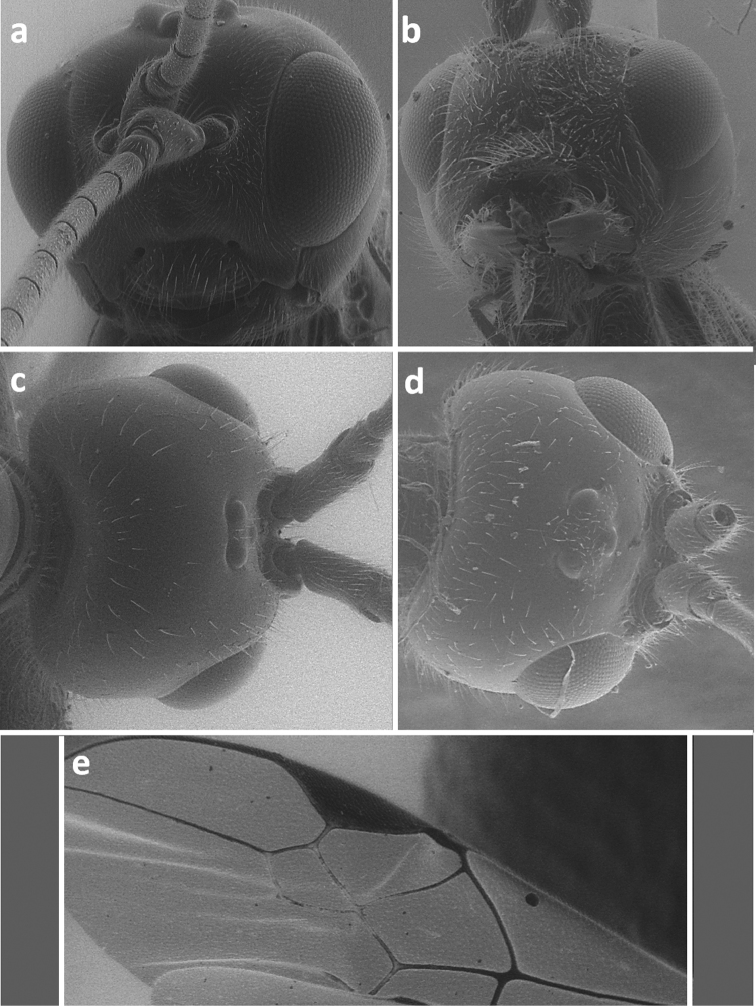
Face in frontal view of *Meteorus corax* (**a**)and *Doryctes striatellus* (**b**); head in dorsal view of *Atanycolus ivanowi* (**c**) and *Doryctes striatellus* (**d**); detail of forewing of *Monochamus corax* (**e**).

**Figure 5.**
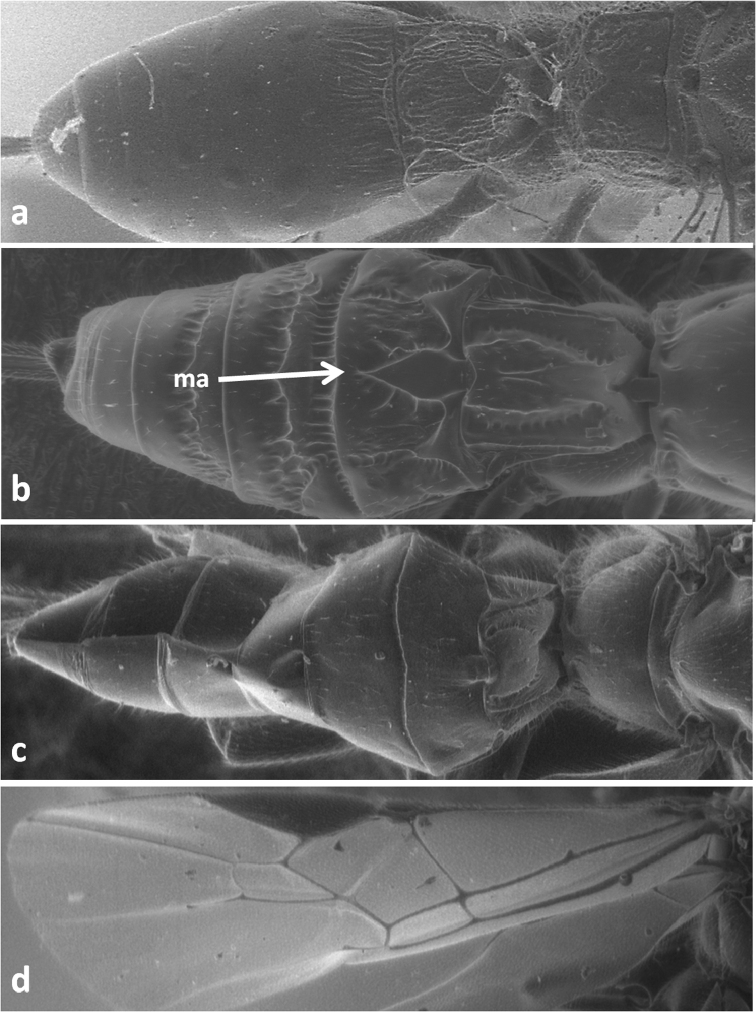
Metasoma in dorsal view of *Doryctes striatellus* (**a**), *Atanycolus ivanowi* (**b**) and *Coeloides sordidator* (**c**); forewing of *Coeloides sordidator* (**d**); ma – mediobasal area.

**Figure 6.**
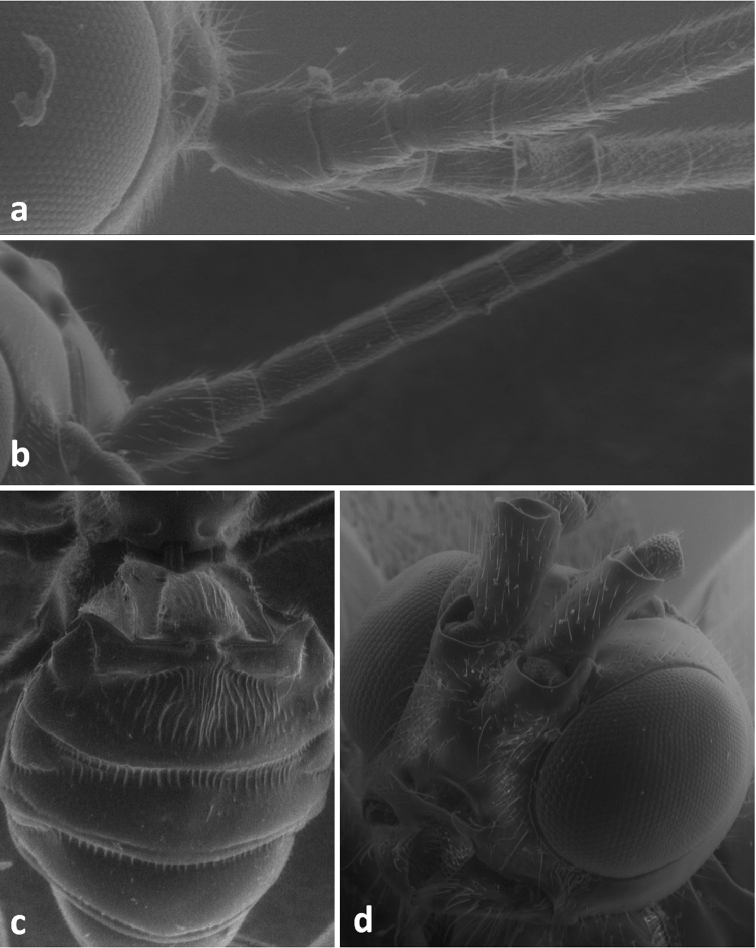
Basal segments of antenna in lateral view of *Coeloides sordidator* (**a**) and *Cyanopterus tricolor* (**b**); metasoma in dorsal view of *Iphiaulax impostor* (**c**); face of *Atanycolus ivanowi* (**d**).

**Figure 7.**
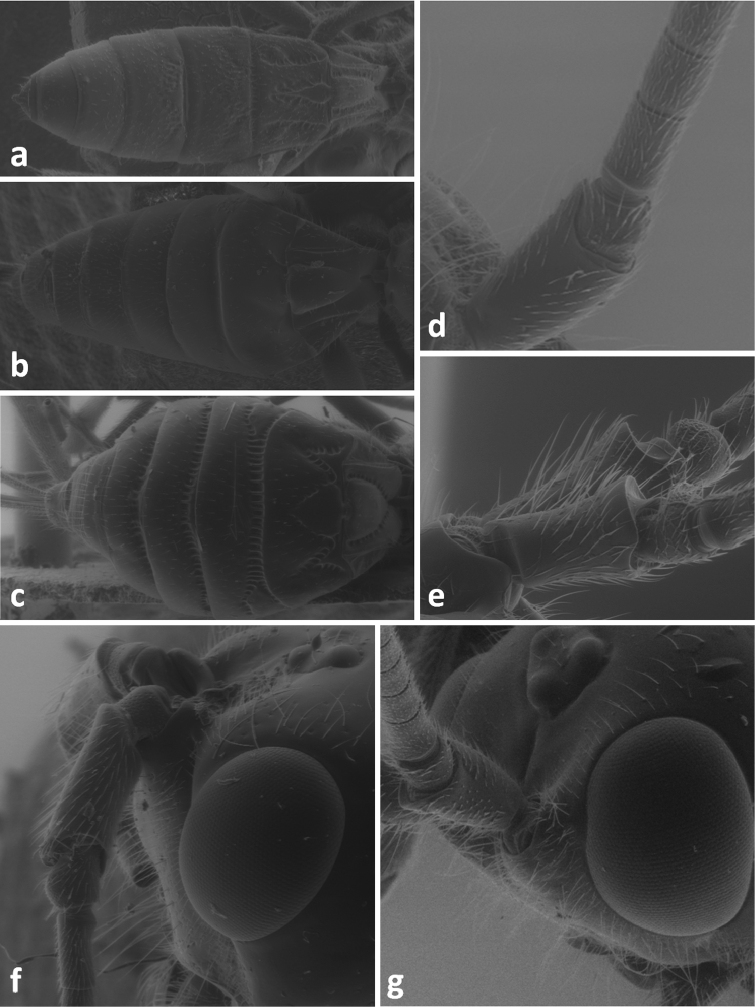
Metasoma in dorsal view of *Atanycolus denigrator* (**a**), *Cyanopterus flavator* (**b**) and *Cyanopterus tricolor* (**c**); scape and pedicel in lateral view of C.* flavator* (**d**) and *Atanycolus denigrator* (**e**); space of head between antennal socket and eye in laterofrontal view of *Atanycolus genalis* (**f**) and *Cyanopterus flavator* (**g**).

## Supplementary Material

XML Treatment for Atanycolus denigrator

XML Treatment for Atanycolus ivanowi

XML Treatment for Cyanopterus flavator

XML Treatment for Doryctes striatellus

XML Treatment for Xorides depressus
